# The New Zealand Thrush: An Extinct Oriole

**DOI:** 10.1371/journal.pone.0024317

**Published:** 2011-09-09

**Authors:** Ulf S. Johansson, Eric Pasquet, Martin Irestedt

**Affiliations:** 1 Department of Vertebrate Zoology, Swedish Museum of Natural History, Stockholm, Sweden; 2 Departement Systématique et Evolution, Muséum National d'Histoire Naturelle, UMR7205-CNRS, F-75231, Paris, France; 3 Molecular Systematics Laboratory, Swedish Museum of Natural History, Stockholm, Sweden; Zoological Society of London, United Kingdom

## Abstract

The New Zealand Thrush, or Piopio, is an extinct passerine that was endemic to New Zealand. It has often been placed in its own family (Turnagridae), unresolved relative to other passerines, but affinities with thrushes, Australaian magpies, manucodes, whistlers, birds-of-paradise and bowerbirds has been suggested based on morphological data. An affinity with the bowerbirds was also indicated in an early molecular study, but low statistical support make this association uncertain. In this study we use sequence data from three nuclear introns to examine the phylogenetic relationships of the piopios. All three genes independently indicate an oriole (Oriolidae) affinity of the piopios, and the monophyly of the typical orioles (*Oriolus*), figbirds (*Sphecotheres*), and the piopios is strongly supported in the Bayesian analysis of the concatenated data set (posterior probability = 1.0). The exact placement of the piopios within Oriolidae is, however, more uncertain but in the combined analysis and in two of the gene trees the piopios are placed basal to the typical orioles while the third gene suggest a sister relationship with the figbirds. This is the first time an oriole affinity has been proposed for the piopios. Divergence time estimates for the orioles suggest that the clade originated ca 20 million years ago, and based on these estimates it is evident that the piopios must have arrived on New Zealand by dispersal across the Tasman Sea and not as a result of vicariance when New Zealand separated from Gondwana in the late Cretaceous.

## Introduction

The New Zealand thrush, or Piopio, was an endemic, but now extinct, passerine on New Zealand. It was widely distributed on both the North and the South Island, from north of Auckland to Steven Island [1]. The birds on the two main islands were morphologically quite similar and have historically often been considered conspecific. They did, however, show some differences in plumage patterns as well as in size and other morphological characters [2] and are today often treated as two different species; the South Island Piopio (*Turnagra capensis*) and the North Island Piopio (*T. tanagra*) [3], [4]. The piopios apparently favoured forest undergrowth and fed on a wide range of food items, including berries, seeds, various invertebrates, eggs and other birds [1]. Of the two species the South Island Piopio was the first to be described in 1787 by Anders Sparrman but in the hundred years that followed it declined from being “common” to virtually extinct by the late 1800s. The North Island Piopio went through a similar decline, and for both species the last confirmed sightings were made at the turn of the century. The primary cause for the decline and final extirpation was apparently caused by predation from introduced predators such as cats, dogs, ferrets, stoats and rats [3].

Historically, the Piopio has often been referred to as the “New Zealand thrush” and was in some 19^th^ century classifications placed in Turdidae [5], [6] (see [2] for a detailed review). However, Buller [7] questioned this association and placed the piopios in their own family, Turnagridae. Later studies have also shown that the thrush-like appearance does not reflect its phylogenetic affinity, but so far no consensus about its actual position within the passerine tree has been reached [4]. For instance, Oliver [1], [8] noted that the palate of the piopios indicated affinity with the Australian magpies (*Gymnorhina tibicen*) or manucodes (*Manucodia*). Mayr and Amandon [9], on the other hand, placed the piopios in their Pachycephalini together with e.g., *Pachycephala*, *Colluricincla*, and *Falcunculus*. However, Mayr and Amandon [9] also placed *Pitohui*, *Oreoica*, *Hylocitrea*, and *Pachycare* in this group, all of which are now considered part of other passerine radiations [10], [11], [12]. Olson et al. [2] examined the osteology, myology and pterolysis of the piopios and concluded that, albeit with considerable conflict among the characters, the piopios were related to the “birds-of-paradise and bowerbird assemblage”. Recent molecular studies, however, have shown that this assemblage is not monophyletic, but rather consists of three separate, unrelated lineages; bowerbirds (Ptilonorhynchidae), birds-of-paradise (Paradisaeidae) and satinbirds (Cnemophilidae) [13].

Nevertheless, a possible relationship with the bowerbirds was also indicated in a molecular study based on 900 base pair (bp) of cytochrome *b* by Chrisitidis et al. [14]. Unfortunately, low bootstrap support for the indicated relationships as well as a limited taxon sampling makes it difficult to draw any firm conclusions from these results. Furthermore, Gibb [15] has recently questioned the accuracy of the sequence used by Chrisitidis et al. [14] and noted that this sequence differs in 45 out of 307 positions (14.7%) compared to a new cytochrome *b* sequence from another individual. In a re-analysis based on 1783 bp of mitochondrial DNA, Gibb [15] instead concluded that the piopios likely belong in the “core Corvoidea” radiation, but were unable to confidently place it in any particular clade within that group.

The majority of the land bird species native to New Zealand, excluding those intentionally or unintentionally introduced by humans, appear to be the result of oversea colonization from the Australian continent [16], [17]. These colonization events range from a few decades (e.g. the Pacific Swallow *Hirundo tahitica* and White-faced Heron *Egretta novaehollandiae*) to many million years ago. But it has also been suggested that some groups, e.g. moas (Dinornithiformes) and New Zealand Parrots (Strigopidae), became isolated when New Zealand broke off from the Gondwanan continent, 80-60 million years ago (mya) [18], [19]. Among passerines, the New Zealand wrens (Acanthisittidae), which represent the first split in the passerine tree, may be another of these groups [13], [20], [21], [22], and it has also been suggested that the New Zealand Wattlebirds (Callaeatidae) became isolated at this event [23] (but see [24]).

The fact that it has been difficult to place the Piopios relative to other groups of passerines could possibly indicate that they are another of these “ancient lineages” that became isolated when New Zealand separated from Gondwana. [23]. The purpose of this study is to resolve the phylogenetic position of the piopios and discern whether this lineage represents one of these potential early divergences or a more recent dispersal.

## Methods

### Taxon sampling, amplification and sequencing

We examined the phylogenetic affinity of the South Island Piopio, *Turnagra capensis*, by analyzing DNA sequences from three nuclear introns; myoglobin intron 2, ornithine decarboxylase introns 6 to 7 (ODC), and glyceraldehyde-3-phosphodehydrogenase intron 11 (GAPDH). DNA was extracted from a foot pad sample obtained from one specimen of *Turnagra capensis* (MNHN 1999-1258), housed in the collections of Muséum National d'Histoire Naturelle, Paris. We used the Qiagen DNeasy Tissue Micro Kit for the extraction following the manufacturer's recommendation, except for that 20 µl of 1 molar DTT (dithiothreitol) was added during the lysis stage and the sample was heated to 72°C for 10 minutes after the buffer AL had been added. To minimize the risk of contamination, extraction where done in a special facility dedicated to the preparation of DNA samples from museum specimen and prior to extraction all equipment and buffers were sterilized with UV light.

Amplification and sequencing of fragmented DNA from old study skins require careful primer design, as target regions usually need to be divided into short, overlapping fragments. We amplified fragments of ca 200–250 bp, generally by using primer combinations that have previously successfully amplified a broad selection of passerine birds, e.g. [25], [26], but for some fragments new primers were also designed. The glyceraldehyde-3-phosphodehydrogenase intron 11 GAPDH was amplified in two fragments using the primer combinations G3Pcora1R [26]/G3PintL1 [27] and G3Pcora1F [26]/G3P14b [27], myoglobin intron 2 was amplified in four fragments using the primer combinations Myo2 [28]/Myo-cora182H [26], Myo-cora159L [26]/Myo344H [22], Myo-TurnF343 (AGT GAC TGG ACA CAA GGG ACA)/Myo-TurnR515 (GCA GAA GCA CTG GGC TCT AT), and Myo-cora491L [26]/Myo3F [29], and ornithine decarboxylase introns 6 to 7 (ODC) was amplified in three fragments using the primer combinations ODintF2 [25]/OD-TurnR1 (CAT GGA AAC TAC AAA AAG ATA CAA AC), OD-TurnF3 (TGT GTG TTT GAT ATG GGA GTA AGT)/OD-TurnR3 (GTA ATA GTC ATT TGA GTT TGA GCT G), and OD-TurnF4 (CTC ATC TAC AGA TGC ACT AAA ATT G)/ODintR4 [25]. We used hot-start touchdown PCR, with annealing temperatures for the first cycles generally just 1–2°C below the melting temperature of the primer with the lowest melting temperature. A representative thermocycling program for a given primer combination started with an initial denaturation at 95°C for 5 min, followed by two cycles of 95°C for 30 s, 59°C for 30 s, 72°C for 60 s, and two sets of cycles, each repeated two times, were the annealing temperature was lowered to 57°C and 55°C, respectively (all other temperatures and intervals identical). The thermocycling program was completed with 34 cycles with the annealing temperature set to 53°C and a final 72°C for 5 min. The extractions, amplifications, and sequencing procedures otherwise followed the procedures described in Irestedt et al. [25].

Our taxon sampling includes a broad selection of oscine birds, including representatives of the bowerbirds, satinbirds, birds-of-paradise, thrushes and whistlers. As preliminary assessments of our first sequences from *Turnagra capensis* indicted an oriole (Oriolidae) affinity, Oriolidae have been particularly densely sampled in the final data set. *Menura novaehollandiae* was used as outgroup as *Menura novaehollandiae* has been found to form the sister clade to all other oscine birds [13]. Voucher and GenBank accession numbers are given in Table 1.

**Table 1:** List of samples, with specimen numbers and GenBank accession numbers.

Species	Clade	MYO	Ref.	ODC	Ref.	G3PDH	Ref.
*Campephaga flava*	Campephagidae	EF052822	[44]	EU380410	[26]	DQ406639	[45]
*Cnemophilus loriae*	Cnemophilidae	EU272107	[46]	EU272126	[46]	EU272096	[46]
*Colluricincla harmonica*	Pachycephalidae	EU273396	[34]	EU273356	[34]	EU273376	[34]
*Coracina cinerea*	Campephagidae	EF052827	[44]	EU380417	[26]	EF052800	[44]
*Dicrurus bracteatus*	Dicruridae	EF052839	[44]	EU272113	[46]	EF052813	[44]
*Eopsaltria australis*	Petroicidae	AY064732	[47]	EF441238	[48]	EF441216	[48]
*Epimachus albertisii*	Paradisaeidae	AY064735	[47]	EU380436	[26]	EU380475	[26]
*Gymnorhina tibicen*	Cractidae	AY064741	[47]	EU272119	[46]	DQ406669	[45]
*Hirundo rustica*	Hirundidae	AY064258	[47]	EF441240	[48]	EF441218	[48]
*Lalage leucomela*	Campephagidae	EF052840	[44]	EU380438	[26]	EF052814	[44]
*Malurus amabilis*	Maluridae	AY064729	[47]	EF441241	[48]	EF441219	[48]
*Manucodia ater*	Paradisaeidae	EU726218	[49]	EU726228	[49]	EU726210	[49]
*Monarcha melanopsis*	Monarchidae	DQ084110	[50]	EU272114	[46]	EU272089	[46]
*Oriolus chinensis*	Oriolidae	EU273404	[34]	EU273362	[34]	EU273382	[34]
*Oriolus flavocinctus*	Oriolidae	EF441258	[48]	EF441243	[48]	EF441221	[48]
*Oriolus oriolus*	Oriolidae	EF052766	[44]	EU273363	[34]	EF052755	[44]
*Oriolus xanthornus*	Oriolidae	AY529929	[51]	EU272111	[46]	DQ406645	[45]
*Pachycephala rufiventris*	Pachycephalidae	EU380510	[26]	EU380445	[26]	EU380481	[26]
*Pericrocotus erythropygius*	Campephagidae	EF052765	[44]	EU380451	[26]	EF052754	[44]
*Picathartes gymnocephalus*	Picathartidae	AY228314	[52]	EF441247	[48]	EF441225	[48]
*Pitohui dichrous*		EU273412	[34]	EU273371	[34]	EU273390	[34]
*Pomatostomus temporalis*	Pomatostomatidae	AY064730	[47]	EF441248	[48]	EF441226	[48]
*Prunella modularis*	Prunellidae	AY228318	[52]	EF441249	[48]	EF441227	[48]
*Ptilonorhynchus violaceus*	Ptilonorhynchidae	AY064742	[47]	EF441250	[48]	EF441228	[48]
*Ptiloprora plumbea*	Meliphagidae	AY064736	[47]	EF441251	[48]	EF441229	[48]
*Saltator atricollis*	Cardinalidae	AY228320	[52]	EF441252	[48]	EF441230	[48]
*Sturnus vulgaris*	Sturnidae	AY228322	[52]	EF441253	[48]	EF441231	[48]
*Sylvia atricapilla*	Sylviidae	AY228323	[52]	EF441254	[48]	EF441232	[48]
*Sphecotheres vieilloti*	Oriolidae	FJ821107	[12]	GQ901707	[35]	GQ901790	[35]
*Terpsiphone viridis*	Monarchidae	AY529939	[51]	EU380458	[26]	DQ406641	[45]
*Turdus philomelos*	Turdidae	DQ466848	[53]	GU358902	[54]	GU359037	[54]
*Turnagra capensis*		JN571533		JN571534		JN571532	
*Vireo flavoviridis*	Vireonidae	EU273417	[34]	EU273374	[34]	EU273394	[34]
OUTGROUP							
*Menura novaehollandiae*	Menuridae	AY064744	[47]	EF441242	[48]	EF441220	[48]

### Phylogenetic analyses

We used Bayesian inference to estimate phylogenetic relationships. The models for nucleotide substitutions used in the analyses were selected for each gene individually by the Akaike Information Criterion using the program MRMODELTEST 2.2 [30] in conjunction with PAUP* [31]. The number of indels was low and the sequences could easily be aligned by eye. The final alignment of the three gene segments included 1744 bp and all gaps were treated as missing data in the analyses.

Posterior probabilities of trees and parameters in the substitution models were approximated with MCMC and Metropolis coupling using the program MRBAYES 3.1.1 [32]. Analyses were performed for each of the individual genes (10 million generations) as well as on the concatenated data set (50 million generations), with trees sampled every 1000 generations. The program AWTY [33] was used to estimate when the chains had reached their apparent target distributions, and trees sampled during the burn-in phase were discarded.

## Results

In total we obtained 1504 bp of nuclear DNA sequences from the *Turnagra capensis* sample (707 bp from myoglobin intron 2, 501 bp from ornithine decarboxylase introns 6 to 7 (ODC), excluding a region of about 100 bp that we were unable to sequence, and 296 bp from glyceraldehyde-3-phosphodehydrogenase intron 11 GAPDH). No mismatches between overlapping fragments were found in any of the target sequences, no heterozygotic sites were found, and none of the sequence fragments turned out to be identical to any other corresponding fragment in any other species checked.

**Figure 1:**
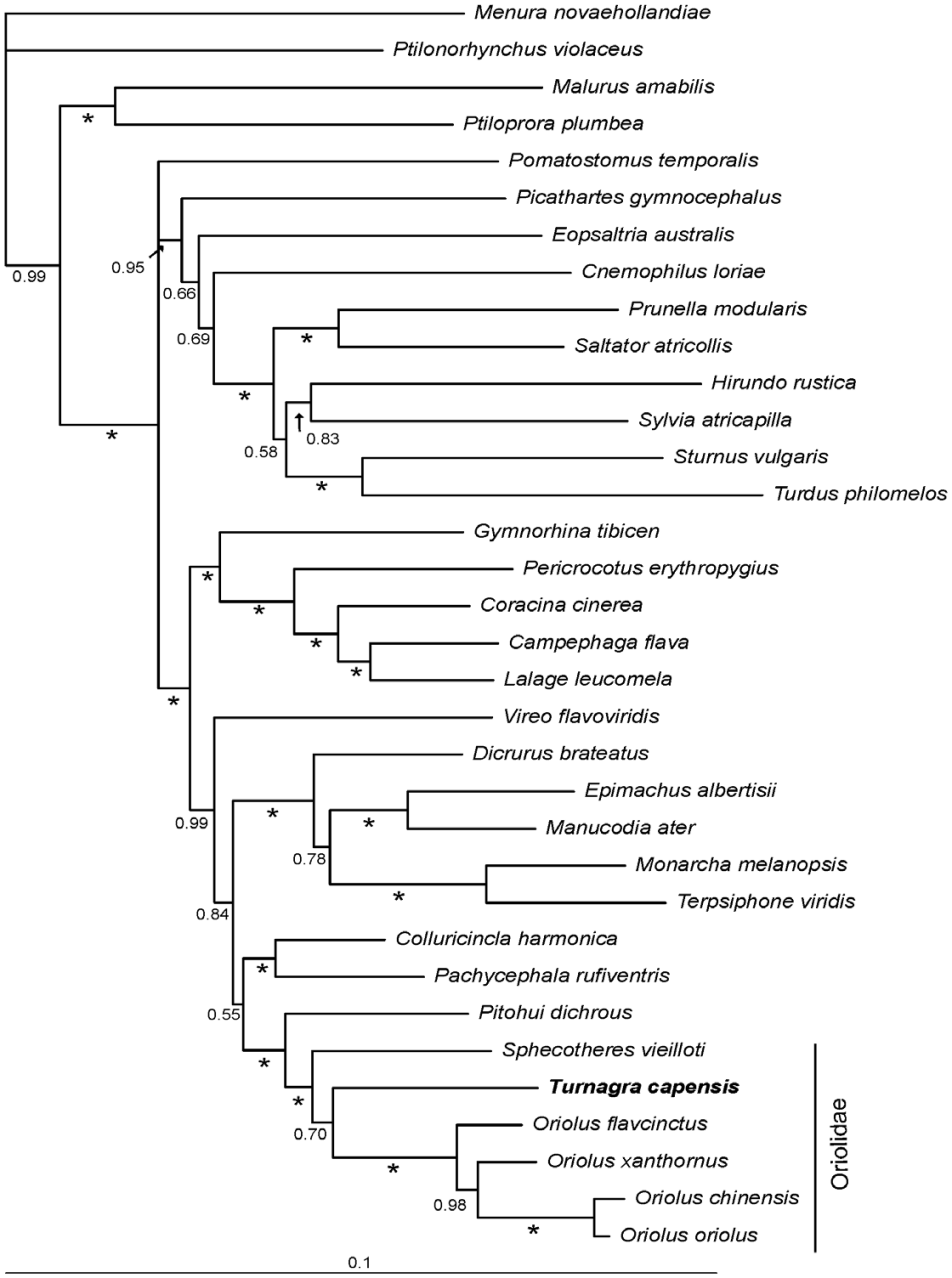
Bayesian consensus tree of the concatenated, mixed model analysis of three nuclear introns (myoglobin, ODC and GAPDH). Posterior probabilities are indicated at nodes. An asterisk * indicates a posterior probability of 1.0. The South Island Piopio (*Turnagra capensis*) is indicated in bold.

The analysis of the concatenated, three-gene data set strongly supports an oriole affinity of the piopios (Fig. 1). The *Turnagra* forms a strongly supported clade (posterior probability [PP] = 1.0) together with the two oriole clades *Oriolus* and *Sphecotheres*. Within this clade the *Turnagra* is placed with weak support (PP = 0.69) as the sister of *Oriolus*. *Pitohui dichrous*, another species with a proposed oriole affinity [34], [35] is placed as the sister group of these three lineages. In this tree, the whistlers (Pachycephalidae), a group to which the piopios sometimes have been assigned, are placed as the sister group of the orioles.

All of the individual gene trees (not shown) indicate an oriole affinity of the piopios, although each gene indicates a slightly different topology. Both myoglobin and G3P place *Turnagra* as the sister of the three included *Oriolus* species, but the two gene trees differ in the placement of *Sphecotheres* and *Pitohui* relative to this group. In the myoglobin gene tree *Sphecotheres* and *Pitohui* are placed as sister taxa, and this clade is in turn placed as the sister group of the *Oriolus*/*Turnagra* clade, but in this gene tree there is also weak support for placing the Yellow-green Vireo *Vireo flavoviridis* with the former two taxa (PP = 0.60). In G3P, neither *Sphecotheres* nor *Pitohui* are placed with the *Oriolus*/*Turnagra* clade, but are placed with different taxa in different parts of the tree. The support values in the G3P gene tree are generally low (PP<0.95), and this tree is, in those parts of importance for this study, basically unresolved due to low posterior probabilities for the indicated relationships. The ODC gene tree is similar to the combined tree in that *Pitohui* is placed basal relative to an *Oriolus*/*Turnagra*/*Sphecotheres* clade, but in this tree *Turnagra* is placed as the sister group of *Sphecotheres* rather than *Oriolus*.

## Discussion

Our data strongly point at an oriole affinity for the piopios and that they are nested within the Oriolidae. This clade consists of the typical orioles *Oriolus* (27 species) and figbirds *Sphecotheres* (3 species) [36], and in our study the piopios are placed with weak support as the sister group of the *Oriolus*, basal relative to other species of that clade (cf. [35]). Our study also confirms that the Hooded Pithoui *Pitoui dichrous* is part of this clade, but rather placed basal relative to the other taxa in this group instead of sisters to the figbirds as indicated by Jønsson et al. [35].

Orioles are distributed in the Eurasian, Afrotropical, Indomalayan and Australasian zoogeographical regions [36]. Most species of orioles in the former regions are bright yellow or red, whereas the figbirds and the other orioles of the Australasian region are mostly drab brown or olive green. The piopios, being olive-grey to olive-brown, were in this respect most similar to the Australasian orioles, and the South Island Piopio had brown streaking on the breast similar to e.g. the Australian Olive-backed Oriole (*Oriolus sagittatus*) as well as females and juveniles of many other oriole species. Very little is known about the biology of the piopios but they appear to have been omnivorous and fed on a wide range of food items, including insects, worms, fruits and berries, much like the orioles. In contrast to the orioles, which rarely descend to the ground for feeding [36], piopios appear to a large extent have foraged on the ground “grubbing with its bill among the dry leaves and other forest debris” [7]. This change in behavior to a more ground-living lifestyle has been rather common among New Zealand birds, and several species of e.g. rails, ducks, parrots and passerines have evolved flightlessness on New Zealand. However, this behavior made them more vulnerable to the introduced predators that arrived in the 19^th^ century and ultimately caused the extinction of many species, including the piopios.

Biogeographical analyses [35] have shown that the Oriolidae likely originated in Australasia, and from there dispersed to other regions. Within the Oriolidae, figbirds, pithouis and the basalmost lineage of *Oriolus* are confined to the Australia, New Guinea and Wallacea; and the placement of the piopios among these groups indicate that the ancestor of the piopios also lived in this region and at some point dispersed to New Zealand. The dating analysis by Jønsson et al [35] indicate that the split between *Sphecotheres/Pithoui* and *Oriolus* took place around 20 Mya, and the earliest split within *Oriolus*, i.e. between the Australasian clade and all other *Oriolus* species, took place around 13 Mya. These estimates provide a rough time frame for the dispersal of the ancestor of the piopios and suggest that the dispersal to New Zealand took place no earlier than approximately 20 Mya.

New Zealand is part of the largely submerged continent Zealandia. This continent, which extends from Caledonia to the subantarctic islands off the cost of New Zealand, was in the Cretaceous above sea level and attached to the large southern hemisphere continent Gondwana. By the end of the Cretaceous (ca 82 Mya) the two continents had begun to separate but may have remained connected in what is now northern Australia until the Early Paleocene (65–61 Mya) or the Early Eocene (ca 52 Mya) [37], [38], [39], [40], [41]. Shortly after Zealandia had separated from Gondwana crustal thinning and stretching resulted in marine transgressions in the Eocene and Oligocene, and by the Late Oligocene most of this region was deep under water [39], [42]. The extent of the Oligocene transgression is unknown but it is clear that much of this region was under water and it has even been suggested that New Zealand was completely submerged around 25 Mya [42], [43].

The phylogenetic position of the piopios within the Oriolidae makes it unlikely that they became isolated on New Zealand when this continent broke off from Gondwana in the Cretaceous. Instead, the divergence time estimate for the Oriolidae [35] suggests that the dispersal took place long after the isolation of New Zealand. Based on these estimates it is likely that the piopios arrived after the Oligocene transgressions, which occurred around 25 Mya, but even though this is a reasonable assumption, these estimates are too crude to establish this with certainty. It is, nevertheless, evident that the piopios add to the list of species that colonized New Zealand once the Tasman Sea had opened rather than being Gondwana relicts.
